# Case Report and Literature Review: Ectopic Thyrotropin-Secreting Pituitary Adenoma in the Suprasellar Region

**DOI:** 10.3389/fendo.2021.619161

**Published:** 2021-03-11

**Authors:** Xiaoxu Li, Binghao Zhao, Bo Hou, Jing Wang, Jianyu Zhu, Yong Yao, Xiaolan Lian

**Affiliations:** ^1^ Department of Neurosurgery, Peking Union Medical College Hospital, Chinese Academy of Medical Science and Peking Union Medical College, Beijing, China; ^2^ Department of Radiology, Peking Union Medical College Hospital, Chinese Academy of Medical Science and Peking Union Medical College, Beijing, China; ^3^ Department of Pathology, Peking Union Medical College Hospital, Chinese Academy of Medical Science and Peking Union Medical College, Beijing, China; ^4^ Key Laboratory of Endocrinology of National Health Commission, Department of Endocrinology, Peking Union Medical College Hospital, Chinese Academy of Medical Science and Peking Union Medical College, Beijing, China

**Keywords:** hyperthyroidism, TSH, MRI, ectopic pituitary adenoma, somatostatin suppression test

## Abstract

Ectopic thyrotropin-secreting pituitary adenoma (ectopic TSH-oma) is a rare disorder that is easily misdiagnosed in clinical work. We report one patient who presented with hyperthyroidism and a suprasellar mass. In this case, preoperative diagnosis of suprasellar ectopic thyrotropin-secreting pituitary adenoma was challenging. A literature review revealed that a total of 11 patients with ectopic TSH-oma were previously reported, and only our one case was diagnosed in the microadenoma stage. Most of the patients with TSH-oma or ectopic TSH-oma were middle-aged. We described ectopic TSH-oma in a child at length. We recommend that ectopic TSH-oma should be considered in the differential diagnosis of thyrotoxicosis syndrome to achieve an accurate, early diagnosis. The somatostatin suppression test and imaging examinations, such as magnetic resonance imaging and positron emission tomography/magnetic resonance imaging, could contribute to the diagnosis. Once the diagnosis was highly suspected, tumor resection could achieve a satisfying long-term outcome in ectopic TSH-oma.

## Introduction

Thyrotropin-secreting pituitary adenoma (TSH-oma) is a rare tumor derived from thyrotrophs in the adenohypophysis. Inappropriate secretion of TSH (ISTSH) syndrome consists of TSH-oma and resistance to thyroid hormone (RTH) syndrome. Since most TSH-oma patients have thyrotoxicosis as the main clinical symptom, it is common for patients to be misdiagnosed and receive anti-thyroid treatment, as shown in previous reports. However, this inappropriate treatment is likely to promote further TSH-oma development, increasing the difficulty of surgery and the possibility of recurrence. TSH-oma is a rare disease, accounting for merely 0.5–2% of all pituitary adenomas (PAs) (1). A literature review revealed that a total of eleven patients with ectopic TSH-oma were previously reported, and only our case here were diagnosed in the microadenoma stage.

## Case Presentation

**Figure 1:**
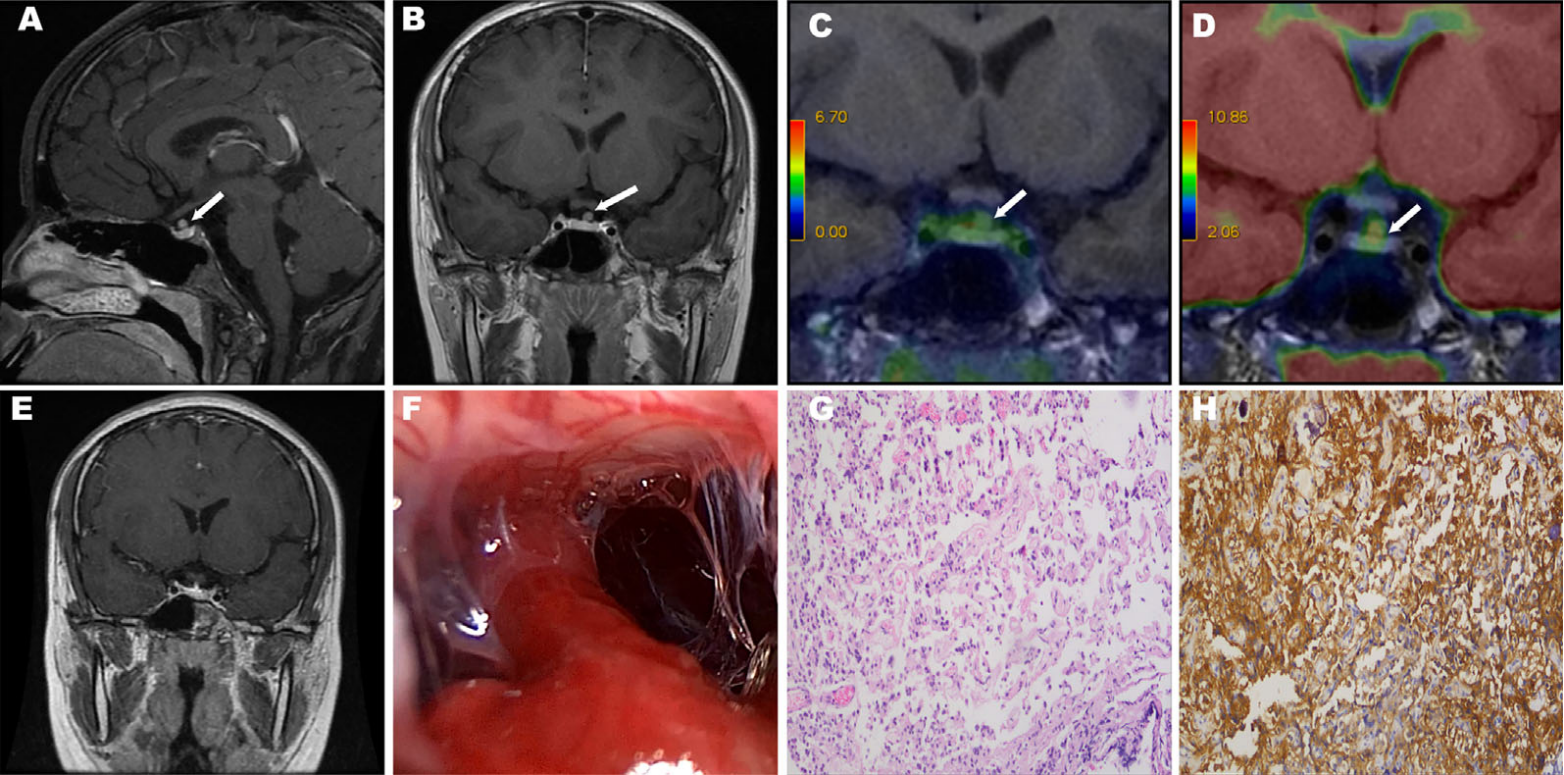
The white arrows show the ectopic tumors. MRI revealed a mass superior of the pituitary gland **(A, B).** PET/MRI: Two markers revealed different degrees of enhancement in the suprasellar region overlapping at the tumor site. Octreotide imaging labeled with 68Ga **(C)**. ^18^F-fluorodeoxyglucose imaging **(D)**. Postoperative MRI revealed that the tumor was completely removed **(E)**. Intraoperative photographs showed the pituitary stalk and sellar diaphragm are intact after tumor resection **(F)**. Histological examination of resected tumor tissue (×100). Hematoxylin-eosin staining reveals pituitary adenoma **(G)**. Immunohistochemical staining shows a positive reaction for thyrotropin **(H)**.

A 10-year-old girl referred to Peking Union Medical College Hospital (PUMCH) presenting with a two-year history of easy hunger, hyperphagia, sweating, heat intolerance, and short temper. Her patients denied a family history of hyperthyroidism and previous interventions for the disease. Physical examination revealed goiter. Cardiac examination revealed sinus arrhythmia. Her blood workup showed a normal level of TSH and elevated FT3, FT4, T3, and T4 levels. The exact values of FT3, FT4, and TSH on admission are 6.00 pg/ml (normal range: 1.80–4.10), 20.29 pg/ml (normal range: 6.1–11.2) and 2.945 uIU/ml (normal range: 0.38–4.34). Anti-thyroglobulin antibody is 20.31 IU/ml (normal range: <115) thyroid peroxidase is 12.61 IU/ml (normal range: <34). Thyroglobulin is 24.90 ng/ml (normal range: 1.40–78.00). Thyrotropin receptor antibody is absence. This case is not associated with excess secretion of other anterior pituitary hormones. Growth hormone(GH) is 0.2 ng/ml (normal range: < 2.0). Adrenocorticotropic hormone (ACTH) is 15.7 pg/ml (normal range: 0–46). Prolactin (PRL) is 9.24 ng/ml (normal range: <30). Sex hormone-binding globulin (SHBG) is 155 nmol/L (normal range: 32.4–128). Thyroid ultrasound revealed multiple cystic thyroid nodules with crystallization, which was consistent with adolescent thyroid manifestations. The right lobe of thyroid gland was 5.0 × 1.8 × 1.5 cm. The left lobe was 5.1 × 1.5 × 1.7 cm. The isthmus was 0.2 cm. There were many echoless areas with regular shape and clear boundary. Inside the areas were high-echo dots with comet tail behind. The larger one was 0.4 × 0.3 cm on the right side and 0.3 × 0.2 cm on the left side. Color Doppler Flow Imaging: no blood flow signal was found. The echo of the rest of glands was uniform. To differentiate the diagnosis of RTH, we performed TR-β gene testing but revealed no mutation. The patient also underwent a somatostatin suppression test, with 0.1 mg octreotide (Sandostatin, Novartis Pharma Stein AG) injected subcutaneously every 8 h. The process of the subcutaneous injection of sandostatin 0.1 mg at 8 am was smooth, but 5 min after injection after next injection at 4 pm, the patient presented with red flush (10 cm×9 cm) and callosity (2 cm×1.5 cm) at the injection site (left upper arm) accompanied by obvious pain and itching. Considering the possibility of allergies, we did not perform the injection of sandostatin at midnight. This patient recovered after the administration of allergy medication. Limited test results showed that the TSH suppression rate was 72.49%. Pituitary dynamic enhanced magnetic resonance imaging (MRI) showed a lesion 4.4×3.1×3.0 (mm) located on the left front of the pituitary stalk, which was suspected as an adenoma in the suprasellar region (**Figure 1**). The challenge of diagnosis was that we could not specify the relationship between mass and hyperthyroidism. And latter positron emission tomography/magnetic resonance imaging (PET/MRI) was carried out, with ^65^Ga-labeled octreotide and ^18^F-labeled FDG as markers. PET-MRI revealed a 4.4 mm nodule-like lesion above the superior border of the pituitary left wing, which could be homogeneously enhanced outside the diaphragma sellae with both markers and had no obvious connection to the anterior pituitary (**Figure 1**). To control the high level of thyroid hormone preoperatively, a therapy of 0.1 mg octreotide (Sandostatin, Novartis Pharma Stein AG) subcutaneous injection combined with cetirizine hydrochloride tablets (Cetirizine, UCB Farchim SA) for skin allergy treatment was conducted for 4 days. Subsequently, she received extended endoscopic transsphenoidal surgery, and the diagnosis of ectopic TSH-oma was confirmed by postoperative pathological examination (**Figure 1**). Immunohistochemistry staining results are as follows. TSH: positive. GH: positive. ACTH: partially positive. Follicle-stimulating hormone (FSH): partially positive. Luteinizing hormone (LH): positive. PRL: negative. Chromogranin A: positive. S-100: negative. Symaptophysin: positive. Ki-67:index<1%. P53: negative. Somatostatin receptor 2: positive. Somatostatin receptor 5: positive. It should be noted that a positive immunohistochemistry finding for one or more pituitary hormones does not necessarily lead to hypersecretion of hormones, which is known as silent PA (1).

**Figure 2:**
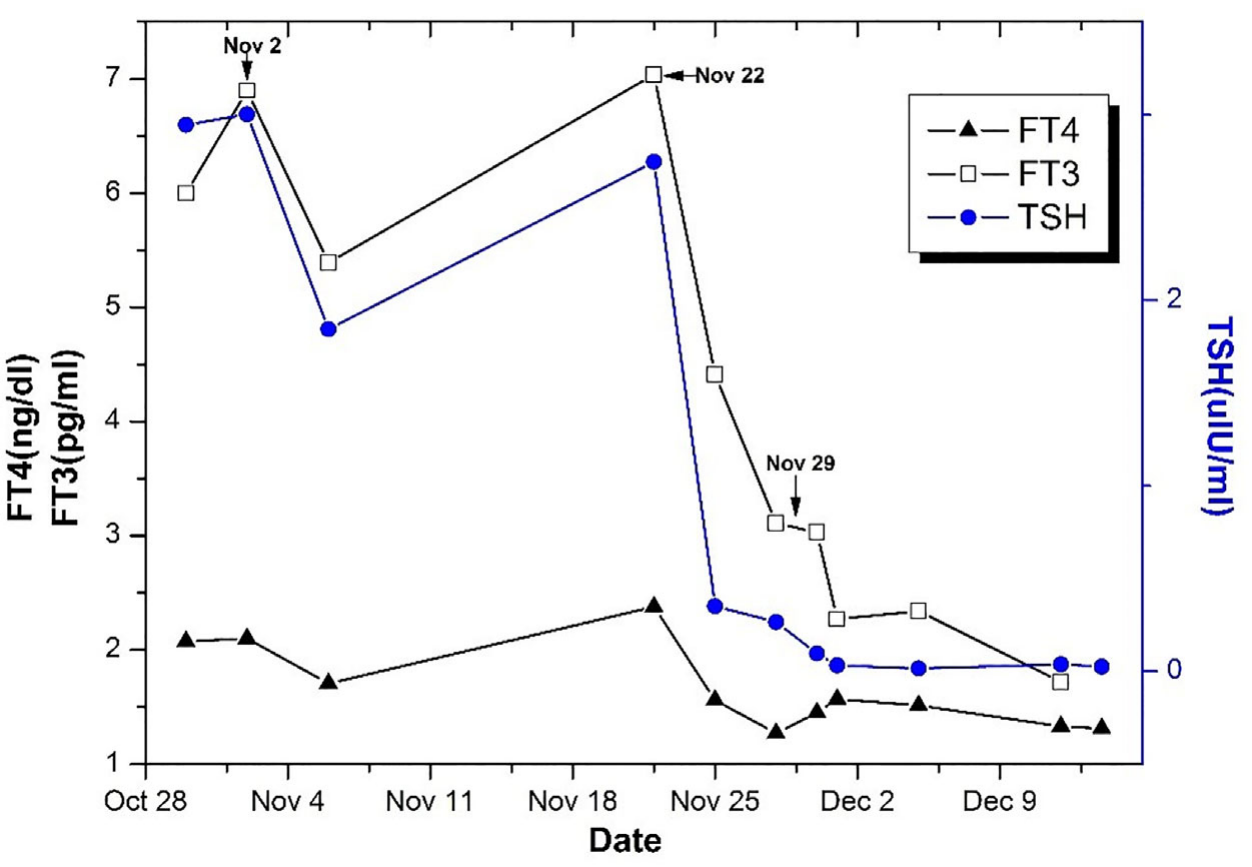
The changes of TSH, FT3, and FT4 levels in the course of treatment. On November 2, Sandostatin 0.1 mg was intramuscularly injected twice. And 0.1 mg Sandostatin was injected Q8H on November 22 for 4 days. The operation was performed on November 29.

Within a week after surgery, a thyroid function test revealed the normalization of FT3, FT4, T3, and T4 levels and a low level of TSH (**Figure 2**). In the follow-up at three months postoperatively, the thyroid function test revealed the normalization of FT3, FT4, T3, T4, and TSH levels, and MRI revealed no tumor remnants. During the follow-up period of 4 years, her clinical presentations improved and achieved long-term remission.

## Discussion

### Ectopic TSH-oma

Pituitary adenomas account for approximately 15% of intracranial tumors (2, 3). However, EPA is a rare disorder, accounting for approximately 0.48% of pituitary adenomas (4). While the exact pathogenic mechanism of EPA has not yet been determined, it is broadly accepted that the tumor originates from the embryonic residues of pituitary cells along the path of migration of Rathke’s pouch (5, 6). Although the origin of EPA is the same as that of the adenohypophysis, EPA refers to pituitary adenomas located completely outside of the sellar turcica without a direct connection to the intrasellar normal pituitary gland or pituitary stalk (7, 8). In a literature review of 85 EPA cases, 72 (85%) EPAs secreted 1 or more hormones, and the most commonly secreted hormone was ACTH (36%), followed by PRL (28%), GH (22%), and TSH (16%) (9). TSH-oma is the rarest form of pituitary adenoma. We performed a literature review to find all reported cases of ectopic TSH-oma with no limitation on the publication date. We searched PubMed using the words “(TSH or thyrotropin) AND (pituitary neoplasm [Mesh]) AND ectopic” and “(TSH or thyrotropin) AND (“pituitary adenoma”) AND ectopic”. Full-text case reports, case series, reviews and original articles written in English were included. Articles were excluded if the cases were not ectopic TSH-oma or the diagnosis was not histologically confirmed. The results of searching PubMed yielded only 11 cases of ectopic TSH-oma (**Table 1**) (6, 10–19). Of all 12 patients, seven (58.33%) were females, and five (41.67%) were males, indicating a female predominance at a ratio of 1.4:1. Except for one patient whose age was unknown, the age of the other patients ranged from eight to 52 years, and most of the patients were middle-aged. The most common location of the ectopic TSH-oma lesion was the nasopharynx (eight cases, 66.67%), followed by the suprasellar region (three cases, 25%) and the sphenoid sinus (only one case so far). Tjörnstrand A proposed that macroadenomas (>10 mm in any dimension) still account for 80–85% of all newly discovered TSH-omas (20). The tumor size data yielded by our ectopic TSH-oma literature review show a similar tendency. Of all the patients with available tumor size data, only our patient had microadenoma (diameter < 1 cm).

**Table 1:** Literature review of reported cases of ectopic TSH-oma.

	Reference	Age at onset/Sex	Location of tumor	Tumor size(mm)	Image examination	Somatostatin suppression test	Surgery
P1	Cooper et al. (10)	45/F	Nasopharynx	NA	MRI, CT	No	Transfacial transmaxillary resection
P2	Pasquini et al. (11)	34/M	Nasopharynx	NA	MRI, Thyroid scintigraphy	No	Endoscopic endonasal surgery
P3	Collie et al. (12)	ND/F	Nasopharynx	20×15	MRI, CT	No	Endoscopic endonasal surgery
P4	Tong et al. (13)	34/F	Nasopharynx and nasal cavity	20×20	MRI, CT, Octreotide scanning, Thyroid ultrasound	Yes	Endoscopic endonasal surgery
P5	Song et al. (14)	40/M	Nasopharynx	19×17	MRI, CT, ECT	Yes	Endoscopic endonasal surgery
P6	Nishiike et al. (15)	36/M	Nasopharynx	14	MRI, CT, PET/CT, Thyroid scintigraphy	No	Endoscopic endonasal surgery
P7	Wang et al. (6)	45/M	Suprasellar	15×12×10	MRI, Thyroid scintigraphy, Thyroid ultrasound	Yes	Endoscopic endonasal transtuberculum
P8	Yang et al. (16)	27/F	Nasopharynx	NA	MRI, Thyroid ultrasound	No	Nasopharynx neoplasm resection
P9	Hanaoka et al. (17)	41/M	Suprasellar	NA	MRI, CT, PET	No	Transcranial approach
P10	Kim et al. (18)	48/F	Nasopharynx	11×8×6	MRI, Radioactive iodine uptake scan, PET/CT, Thyroid ultrasound	No	Endoscopic endonasal approach
P11	Ortiz et al. (19)	52/F	Sphenoid sinus	24×23×13	MRI, CT, Thyroid ultrasound	Yes	Endoscopic transsphenoidal surgery twice
P12	Present case	8/F	Suprasellar	4.4×3.1×3.0	MRI, PET/MRI, Somatostatin receptor imaging, Thyroid and cervical lymph node ultrasound	Yes	Endoscopic transsphenoidal surgery

NA, not available; PET/CT, positron emission tomography/computed tomography; PET, positron emission tomography; CT, computed tomography; MRI, magnetic resonance imaging; ECT, emission computed tomography; PET/MRI, positron emission tomography/magnetic resonance imaging.

### Diagnosis of Ectopic TSH-oma in the Sellar Region

Most patients with TSH-oma present with thyroid dysfunction, which was initially misdiagnosed as Graves’ disease, and some patients even received inappropriate treatment with thyroidectomy or radioiodine thyroid ablation (1, 21). A similar phenomenon was found in another reported case of ectopic TSH-oma, and misdiagnosis led to delayed or inappropriate treatment that worsened the disease. Therefore, it is of clinical value for us to summarize existing experience and clinical evidence to facilitate accurate, early diagnosis. Except for the reported cases of ectopic TSH-oma in the nasopharynx (8/15), in which the patients presented with nasal obstruction symptoms, there were no reported cases of special symptoms in cases of ectopic TSH-oma in the sellar region. Similarly, the coexistence of elevated serum FT4/FT3 and a lack of TSH suppression in a patient with manifestations of hyperthyroidism, which is called central hyperthyroidism, could exclude Graves’ disease (1). Although the most likely cause of central hyperthyroidism is TSH-oma (21), some patients with RTH syndrome might present with hyperthyroidism, which could be confusing. High levels of SHBG in TSH-oma and normal levels in RTH syndrome could reflect the expected action of excess T4 and T3 on hepatic SHBG production in hyperthyroidism and resistance to hormone action in RTH syndrome. TRβ gene analysis could help to exclude RTH syndrome, as genomic TRβ mutations have been detected in patients with RTH syndrome but not TSH-oma (1). The somatostatin suppression test contributes to the diagnosis and differential diagnosis of TSH-oma. Because the somatostatin receptor is expressed on the surface of TSH-oma cells (22), the secretion of TSH could be inhibited after somatostatin analog injection in patients with TSH-oma. The TSH suppression rate was calculated by the proportion of the largest decrease in the initial TSH level. Similarly, we observed an obvious inhibitory effect of somatostatin analogs in cases of ectopic TSH-oma.

Another key link in the diagnosis and differential diagnosis of ectopic TSH-oma is imaging examinations. Computed tomography (CT) and MRI could contribute to detecting a pituitary lesion in TSH-oma patients (23). Due to the high resolution of soft tissue, we routinely performed pituitary dynamic MRI for the diagnosis of ectopic TSH-oma at PUMCH. Pituitary MRI has a high sensitivity for the identification of a sellar or parasellar lesion (24). Pituitary dynamic MRI can clearly present the location, size, edge and extent of the tumor, and it could also show the condition of the pituitary gland and the relationship between the tumor and the normal pituitary gland and pituitary stalk, thus providing significant guidance for preoperative localization and the surgical strategy. It has been proposed that the presentation of EPA on MRI is a mass with equal T1 and T2 signals with no connections to the pituitary gland or pituitary stalk, with enhanced scanning revealing no obvious enhancement or nonuniform enhancement (25). It has also been reported that a pituitary lesion is identified by MRI in 20% of RTH syndrome cases, which may suggest the coexistence of pituitary incidentaloma and RTH syndrome (26). Additional imaging examinations, such as positron emission tomography/computed tomography (PET/CT) and PET/MRI, could contribute to the evaluation of the tumor location. ^18^F-fluorodeoxyglucose (FDG) PET/CT is proposed to be positive in 60–67% of cases in studies involving pituitary adenomas (27, 28), which can be meaningful for detecting adenomas, especially when the MRI findings are unclear. Ishizaki et al. proposed that PET/CT revealed increased FDG uptake in EPA patients (4). ^68^Ga DOTA-labeled somatostatin analogs, such as ^68^Ga DOTATATE, have been reported as somatostatin receptor imaging agents in PET/CT and PET/MRI to detect the presence of neuroendocrine tumors (18). Especially for well-differentiated neuroendocrine tumors, ^68^Ga-DOTATATE PET has been proposed to have improved lesion detection compared with Octreoscan, ^18^F-FDG PET/CT and PET/MRI (29). Zhao et al. confirmed different degrees of uptake of ^68^Ga-DOTATATE and ^18^F-FDG PET/CT in pituitary tissue and adenoma tissue, and the utilization of both would contribute to the differentiation of recurrent or residual pituitary adenoma and the remaining normal pituitary tissue after transsphenoidal tumor resection (30). Two markers revealed overlapping enhancement in the suprasellar region where the tumor was, which further supported the diagnosis of EPA (**Figure 1**).

### Treatment of Ectopic TSH-oma

Transsphenoidal pituitary surgery has been regarded as the first choice for treatment for TSH-oma (31). All 12 patients underwent surgery for ectopic TSH-oma removal. The key component of preoperative preparation is reducing the level of thyroid hormone to control preoperative thyrotoxicosis and prevent a thyroid storm, which is an acute and lethal condition that can be caused by surgical trauma. Fujio et al. reported 1 case of postoperative thyroid storm secondary to hyperthyroidism induced by TSH-oma (32). Noriaki Fukuhara et al. proposed that short-term preoperative octreotide administration could shrink TSH-oma and normalize hormone levels (33). Tong et al. proved that octreotide could inhibit TSH secretion by TSH-oma tissue *in vitro* (13). Similarly, in our case, the patient achieved obvious improvements in hormone levels after the administration of somatostatin analogs before the operation. At PUMCH, anti-thyroid drugs are not commonly the first drug choice. However, in some situations where patient is not sensitive to somatostatin, we use them to decrease preoperative hormone levels. It has been proposed that the possibility of the presence of invasive macroadenomas is exceedingly high in patients with a history of thyroid ablation (21), which places a greater demand on surgeons and increases the risk of surgery. Therefore, diminishing the misdiagnosis of ectopic TSH-oma is significant. Because of the special locations of SPAs, our case subsequently underwent extended endoscopic TSS. Endoscopic endonasal transtuberculum sellar surgery has been reported for the treatment of suprasellar tumors with increasing indications (6).

## Conclusions

Currently, experience in the diagnosis of ectopic TSH-oma, especially those in the sellar region, is limited, as it is rare and has no special symptoms. We were the first to report ectopic TSH microadenoma worldwide. Ectopic TSH-oma should be considered in the differential diagnosis of thyrotoxicosis syndrome to achieve an accurate early diagnosis. To specify the tumor characteristics and the relationship with thyrotoxicosis, the inhibitory effect of somatostatin analogs in the somatostatin suppression test contributes to the differential diagnosis. Imaging examinations such as MRI could clearly reveal the mass without connections to the pituitary gland or pituitary stalk. PET could help to identify the character of mass. Once the diagnosis was highly suspected, tumor resection could achieve a satisfying outcome in ectopic TSH-oma.

## Data Availability Statement

The raw data supporting the conclusions of this article will be made available by the authors, without undue reservation.

## Ethics Statement

Prior written permission was obtained from the patient for treatment as well as for the preparation of this manuscript and for publication.

## Author Contributions

XLi and BZ wrote the manuscript. BH collected the ata. JZ, YY, and XLian provided suggestion during the diagnosis and treatment in this case. All authors contributed to the article and approved the submitted version.

## Funding

This work was supported by the Chinese Academy of Medical Sciences Innovation Fund for Medical Science (CAMS-2016-I2M-1-002).

## Conflict of Interest

The authors declare that the research was conducted in the absence of any commercial or financial relationships that could be construed as a potential conflict of interest.

## Abbreviations

TSH-oma, Thyrotropin-secreting pituitary adenoma; PUMCH, Peking Union Medical College Hospital; ISTSH, Inappropriate secretion of TSH; RTH, Resistance to thyroid hormone; PA, Pituitary adenoma; EPA, Ectopic pituitary adenoma; TSH, Thyrotropin; FT4, Free tetraiodothyroxine; FT3, Free triiodothyroxine; ACTH, Adrenocorticotropic hormone; PRL, Prolactin; FSH, follicle stimulating hormone; GH, Growth hormone; T3, Triiodothyronine; T4, Thyroxine; LH, Luteinizing hormone; IGF-1, Insulin-like growth factor-1; SHBG, Sex hormone-binding globulin; MRI, Magnetic resonance image; TSS, Transsphenoidal surgery; CT, computed tomography; PET, Positron emission tomography; FDG, ^18^F-fluorodeoxyglucose.
